# Anti-diabetic effects of the soluble dietary fiber from tartary buckwheat bran in diabetic mice and their potential mechanisms

**DOI:** 10.29219/fnr.v65.4998

**Published:** 2021-01-08

**Authors:** Weijing Wu, Zaigui Li, Fei Qin, Ju Qiu

**Affiliations:** 1 Department of Public Health and Medical Technology, Xiamen Medical College, Xiamen, Fujian, China; 2 Fujian Provincial Key Laboratory of Food Microbiology and Enzyme Engineering, Xiamen, Fujian, China; 3 College of Food Science and Nutritional Engineering, China Agricultural University, Haidian, Beijing, China; 4 Institute of Food and Nutrition Development, Ministry of Agriculture and Rural Affairs, Haidian, Beijing, China

**Keywords:** soluble dietary fiber, tartary buckwheat, glucose metabolism, lipid metabolism, short-chain fatty acids, AMP-activated protein kinase

## Abstract

**Background:**

Tartary buckwheat has beneficial effects on glucose and lipid metabolism of patients with type 2 diabetes mellitus. However, the physiological effects of a soluble dietary fiber (SDF) from tartary buckwheat have rarely been studied, especially *in vivo*.

**Objective:**

This study aimed to examine the hypoglycemic and hypolipidemic effects of SDF from tartary buckwheat bran on high-fat diet/streptozotocin-induced diabetic mice.

**Design:**

The SDF of tartary buckwheat bran was collected according to the Association of Official Analytical Chemists method 991.43. Diabetic mice were treated with high-fat diets supplemented with 0.5, 1, and 2% SDF for 8 weeks. Parameters related to glucose and lipid metabolism and relevant mechanisms, including the excretion of short-chain fatty acids and the glycemic signaling pathway in the liver, were investigated. In addition, the structural characterization of a purified polysaccharide from SDF of tartary buckwheat bran was illustrated.

**Result:**

Supplementation with SDF in the diet resulted in reduced levels of fasting blood glucose, improved oral glucose tolerance, increased levels of liver glycogen and insulin, as well as improved lipid profiles in both the serum and liver, in diabetic mice. The amelioration of glucose and lipid metabolism by SDF was accompanied by an increase in the short-chain fatty acid levels in the cecum and co-regulated by hepatic adenosine-5′-monophosphate-activated protein kinase (AMPK) phosphorylation. A neutral tartary buckwheat polysaccharide with an average molecular weight of 19.6 kDa was purified from the SDF, which consisted mainly of glucose with α-glycosidic bonds.

**Conclusions:**

The SDF of tartary buckwheat bran exhibits hypoglycemic and hypolipidemic effects in diabetic mice, contributing to the anti-diabetic mechanisms of tartary buckwheat.

## Popular scientific summary

- The soluble dietary fiber from tartary buckwheat bran improved glucose and lipid metabolism in diabetic mice.
- This soluble dietary fiber increased the short-chain fatty acid levels in cecum and promoted phosphorylation of adenosine-5′-monophosphate-activated protein kinase in liver.
- The structural characterization of a purified polysaccharide from the soluble dietary fiber of tartary buckwheat bran was illustrated.

Diabetes is a complex disease that causes elevated blood glucose levels. It involves either a deficiency in insulin secretion, resistance to insulin action, or both, and can also be associated with lipid disorder, cardiovascular disease, retinopathy, and other pathologies (1). Higher intake of fiber has been shown to lower the risk of type 2 diabetic mellitus (T2DM) (2). Pseudo-cereals have high levels of dietary fiber (3), and they show many beneficial, physiological activities (4, 5). The soluble dietary fiber (SDF) from pseudo-cereals can be considered an alternative functional product for hyperglycemia and hyperlipidemia.

Tartary buckwheat (*Fagopyrum tataricum*) is a functional grain that can effectively improve the glucose and lipid metabolism of T2DM patients (6). Most studies have ascribed these promising beneficial health effects to tartary buckwheat’s flavonoids, proteins, and D-chiro-inositol. It was reported that tartary buckwheat from Islek contains 24.76% of total dietary fiber in bran fraction (7). The content of total dietary fiber in bran was higher than that in flour (7). However, few studies have revealed its physiological effects. The purified non-starch polysaccharide from tartary buckwheat exhibits *in vitro* inhibitory activity against α-D-glucosidase (8), indicating its potential anti-diabetic properties. Nevertheless, the *in vivo* physiological functions of SDF remain to be studied.

This study, therefore, aimed to examine the hypoglycemic and hypolipidemic effects of SDF from tartary buckwheat bran on high-fat diet/streptozotocin-induced diabetic mice. Parameters by which the relevant mechanisms were investigated include the excretion of short-chain fatty acids (SCFAs) and the glycemic signaling pathway in the liver.

## Materials and methods

### Materials

The tartary buckwheat (Xiqiao No. 2) was collected from Liangshan, Sichuan Province, China. Alcalase 2.4 L (2.4 U/g), acetic acid, propionic acid, and butyric acid were purchased from Sigma-Aldrich Chemicals (Shanghai, China). Amyloglucosidase (100 IU/mg) and heat-stable α-amylase were purchased from J & K Chemicals (Beijing, China). Commercial assay kits for glucose, total triglycerides (TG), total cholesterol (TC), low-density lipoprotein cholesterol (LDL-c), high-density lipoprotein cholesterol (HDL-c), aspartate aminotransferase (AST), alanine aminotransferase (ALT), and glycosylated serum protein (GSP) were purchased from Jiancheng Bioengineering Institute (Nanjing, China). The mouse insulin Enzyme-linked immunosorbent assay (ELISA) assay kit was purchased from Cusabio Life Science (Wuhan, China). Primary antibodies against β-actin, adenosine-5′-monophosphate-activated protein kinase (AMPKα), phospho-AMPKα (Thr172), and the secondary antibody of anti-rabbit IgG were purchased from Cell Signaling Technology (MA, USA). The polyvinylidene fluoride (PVDF) membrane and enhanced chemiluminescence (ECL) Western horseradish peroxidase (HRP) substrate were obtained from Millipore (MA, USA). All other chemicals purchased were of standard analytical grade.

### Determination and preparation of soluble dietary fiber

The SDF was extracted from tartary buckwheat bran. The SDF in bran was prepared and their content was determined according to the Association of Official Analytical Chemists (AOAC) official method 991.43 (total, including soluble, insoluble dietary fiber in foods, enzymatic-gravimetric method, Mes-Tris buffer). In brief, tartary buckwheat bran was defatted with 85% ethanol twice (1:5, w/v, 85°C, 1 h) to remove most lipids, flavonoids, and soluble low-molecular-weight carbohydrates, and then dried at 60°C overnight. The defatted flour was suspended in distilled water (1:10, w/v) and boiled for starch gelatinization (95°C, 15 min). Thermally stable α-amylase was added to hydrolyze the starch (95°C, 30 min) up to the negative iodine test. Subsequently, alcalase was added for protein degradation (60°C, 4 h, pH 7.5). Papain and trypsin were added to further hydrolyze the protein residues. The suspension was then incubated with amyloglucosidase (55°C, 2 h, pH 4.5). Following this, the mixture was neutralized and heated to terminate enzymatic hydrolysis (80°C, 15 min). The supernatant was collected by centrifugation at 5,500 rpm for 20 min (Avanti JXN-26, Beckman Coulter, USA) and mixed with ethanol at a 1:4 ratio. The resulting precipitate was obtained by centrifugation and was lyophilized to obtain the SDF fraction. The procedure was repeated to obtain sufficient SDF for further study.

### Purification and structural analysis of polysaccharide from SDF

#### Extraction of SDF and purification of polysaccharide from SDF

The SDF was dissolved in distilled water and then centrifuged to remove the insoluble residues (5,500 r/min, 10 min). The supernatant was purified through a Diethylaminoethyl (DEAE) Sepharose CL-6B column (3.5 × 30 cm) eluted with 0, 0.6, and 1.2 M NaCl in 20 mM Tris-HCl buffer (pH 7.0) at a flow rate of 5 mL/min. The total carbohydrate content was measured using the phenol-sulfuric acid colorimetric method at 490 nm. The protein in the elute was detected by A280 nm. Two fractions from the elution steps, tartary buckwheat polysaccharide 1 (TBP1) and tartary buckwheat polysaccharide 2 (TBP2), were concentrated by rotary evaporation, dialyzed, and lyophilized. Further purification of TBP1 was performed on a Sepharose CL-6B column (2.5 × 55 cm) eluted with deionized water at a flow rate of 1.5 mL/min.

#### Molecular weight distribution of TBP1

The average molecular weight of TBP1 obtained above was measured by online multi-angle laser light scattering (DAWN EOS, Wyatt Technology Inc., USA) combined with gel permeation chromatography according to the previous study (9).

#### Monosaccharide composition analysis of TBP1

The monosaccharide compositions of TBP1 were determined by ion-exchange chromatography (ICS-3000, DIONEX, USA) with a pulsed amperometric detector according to the previous study (9).

#### Fourier transform infrared spectroscopy

The TBP1 (2 mg) was mixed and grounded with KBr and pressed into a film under the pressure of oil pump. FT-IR spectra were recorded with a Bruker tensor 27 Fourier transform infrared spectrometer (FT-IR) (Germany).

#### Nuclear magnetic resonance

High-resolution nuclear magnetic resonance (NMR) spectroscopy was performed using a Bruker Avance III spectrometer (Germany), which is operated at 400 MHz for ^1^H NMR, ^13^C NMR, ^1^H-^13^C HSQC spectra, and ^1^H-^1^H HSQC spectra, respectively, at 25°C in sample tubes with a diameter of 3 mm.

### Animals experiment

Four-week-old male C57BL/6 mice (*n* = 50, Certificate No. 2015000543572) were obtained from Beijing HFK Bioscience Co. Ltd (Beijing, China). After acclimatization for 1 week, the mice were divided into a healthy group and a diabetic model group. The healthy group was given a standard diet during the entire course of the experiment. The induction of the diabetic model was based on previous research (10) with some modifications. The diabetic mice were fed a high-fat diet (45% kcal of energy from fat, Research diets D12451, Table 1) for 5 weeks, and then were subjected to fasting for 18 h (free access to water). They were then given an intraperitoneal injection of streptozotocin (STZ) (120 mg/kg, citrate buffer, pH 4.5) and subjected to a further 2-h fasting period. The diabetic mouse model was confirmed by fasting blood glucose (FBG), which was maintained above 11.1 mM continuously for 2 weeks after STZ injection. The eligible diabetic mice were subdivided into four groups according to weight and FBG; these included the diabetic model group (10 mice, 3–4 mice/cage), the low-dose SDF group (0.5%), the middle-dose SDF group (1%), and the high-dose SDF group (2%) (*n* = 9 mice in each SDF group, three mice/ cage). All mice were allowed free access to experimental diets for 8 weeks. Food intake and water intake were measured every day. The glucose levels of mice were measured every 2 weeks. The use of animals and experimental methods was in compliance with the National Institutes of Health Guidelines for the Care and Use of Laboratory Animals.

**Table 1:** Ingredients and chemical composition of experimental diets

Ingredient g/kg diet	High-fat diet	Soluble dietary fiber (SDF)		
		0.5%	1.0%	2%
Maize starch	84.8	83.9	83.0	81.1
Cellulose	58.3	57.6	57.0	55.7
Maltodextrin	116.5	115.3	114.0	111.5
Sucrose	201.4	199.2	197.0	192.6
**SDF**	**0**	**5.0**	**10.0**	**20.0**
Soybean oil	29.2			
Lard	206.8			
Casein	233.1			
Cystine	3.5			
Mineral mix	52.4			
Vitamin mix	11.6			
Choline Bitartrate	2.3			
Red dye	0.1			
Total (g)	1000.0			

#### Oral glucose tolerance test

One day before the end of experiment, the mice were fasted for 14 h, followed by an oral administration of 20% (w/v) glucose solution at a dose of 2 g/kg. Blood was collected from the tail vein at 0, 30, 60, 90, and 120 min after administration of glucose. The blood glucose levels were determined using a commercial assay kit and recorded as BG_0_, BG_30_, BG_60_, B_90_, and B_120_. The area under the curve (AUC) was calculated as follows:

AUC _0–120min Glu_ (mM × *h*) = 0.25 × BG_0_ + 0.5 × (BG_30_ + BG_60_ + BG_90_) + 0.25 × BG_120_.

At the end of experiment, the mice were fasted for 14 h before being sacrificed by cervical dislocation after anesthesia. Blood samples were collected from the orbital venous plexus. The serum was separated by centrifugation (3,000 r/min, 10 min) after coagulation at room temperature for 30 min and was maintained at −20°C. The related parameters in the serum were measured using a commercial assay kit. The organs were rinsed with 0.9% NaCl solution, immediately frozen in liquid nitrogen, and stored at −80°C until analysis.

### Assay of hepatic lipid analysis

The liver tissue was weighed and homogenized (IKA^®^ T10) with a 0.9% NaCl solution (1:9 w/v) according to the manufacturer’s instructions. The TC and TG contents in the homogenate were determined using a commercial assay kit.

### Assay of total liver glycogen

The frozen liver samples were weighed, dissolved in a 30% KOH solution, and boiled for 20 min. Glycogen was precipitated by adding ethanol. After centrifugation (1,700 × g, 10 min, 4°C) and decanting of the supernatant, the pellet was resolved in distilled water. The glycogen content was determined using the phenol–sulfuric acid method.

#### Extraction and analysis of SCFAs in the cecum

The measurement of SCFA levels in the cecum was performed as described in a previous study (11). In brief, the cecal content was mixed with acidified water (pH 1.5) with H_2_SO_4_ (1:5 w/v) at 4°C for 30 min and vortexed every 10 min. Then, the mixture was centrifuged at 10,000 r/min for 20 min at 4°C. The supernatant was transferred into a new tube and mixed with an equal volume of ethyl ether. After centrifugation, the ethyl ether extract was filtered through a 0.22 μM filter. Cecal SCFAs were determined by gas chromatography (Agilent 7890B GC system equipped with a flame ionization detector). The SCFAs were separated on an HP INNO-WAX (ID 25 mm, length 30 m, 0.25 μm film thickness, J&W scientific, USA) using the following program. The initial temperature was 100°C. It was raised to 170°C by 6°C/min, increased to 230°C by 20°C/min, and held at 230°C for 2 min. The injection port was operated at 250°C. The detector temperature was 280°C. The carrier gas was nitrogen at 25 mL/min. Sample quantification was carried out using the standard SCFAs (acetic, propionic, and butyric acid) at concentrations of 0.1–2 mg/mL.

### Western blotting

Total protein was extracted from the liver tissue using radio-immunoprecipitation assay (RIPA) lysis buffer. Aliquots of the liver protein extract (25 μg protein/lane) were separated by 10% sodium dodecyl sulfate polyacrylamide gel electrophoresis (SDS-PAGE) and transferred onto a PVDF membrane. The PVDF membrane was blocked with 5% bovine serum albumin in TBS-Tween buffer for 1 h and incubated with the first antibody at 4°C overnight. β-Actin was used as the control to ensure a consistent amount of loaded protein. The membrane was washed three times in Tris-buffered saline-Tween and further incubated with secondary antibody at room temperature for 1 h. Protein expression was detected using an ECL detection kit, which was exposed by Bio-Rad ChemiDoc XRS+ Imager (USA). Densitometric analysis was performed using Image J software.

### Statistical analysis

Values are expressed as the mean ± standard error of the mean (SEM). Statistical differences among groups were evaluated by one-factor analysis of variance (ANOVA) and Duncan’s test for post hoc analysis. Data were considered to be statistically significant at *P* < 0.05. The other statistical evaluations were performed using Student’s *t*-test.

## Results

### SDF ameliorated glucose metabolism in diabetic mice

#### SDF reduced FBG and glycosylated serum protein (GSP)

The changes in levels of FBG and GSP are shown in Table 2. The FBG level of the diabetic model increased from 25.58 ± 1.33 mM to 28.35 ± 0.16 mM during the 8-week treatment, which was a significantly larger change than that of the healthy group. Prior to treatment, FBG showed no significant difference among all diabetic groups (*P* > 0.05). There were no significant differences in food intake and body weight among the diabetic and SDF groups. After 8-week treatment, FBG levels in the 0.5, 1, and 2% SDF groups were significantly reduced by 18.0, 18.3, and 16.6%, respectively (*P* < 0.05). The change in the level of GSP was similar to that of FBG. Treatment with SDF at doses of 0.5, 1, and 2% resulted in significant GSP reductions by 12.2, 6.8, and 11.6%, respectively (*P* < 0.05). However, the dosage effects of SDF on FBG and GSP were not significant.

**Table 2:** Weekly fasting blood glucose (FBG) change and final glycated serum protein (GSP) level

Groups	FBG (mM)					GSP (mM)
	0 week	2nd week	4th week	6th week	8th week	8th week
Healthy	6.7 ± 0.21^a^	6.57 ± 0.10^a^	5.63 ± 0.17^a^	7.03 ± 0.43^a^	7.6 ± 0.43^a^	1.21 ± 0.11^a^
Diabetic model	25.58 ± 1.33^b^	26.0 ± 0.84^b^	27.18 ± 0.63^b^	26.74 ± 0.47^b^	28.35 ± 0.16^b^	3.18 ± 0.26^b^
Soluble dietary fiber (SDF) 0.5%	25.00 ± 0.81^b^	24.16 ± 0.93^b^	25.44 ± 0.56^c^	23.43 ± 0.70^c^	23.26 ± 0.21^c^	2.77 ± 0.10^c^
SDF 1%	25.41 ± 0.79^b^	24.4 ± 1.12^b^	23.89 ± 0.57^c^	24.00 ± 0.59^c^	23.19 ± 0.28^c^	2.94 ± 0.17^c^
SDF 2%	25.56 ± 1.13^b^	24.54 ± 0.98^b^	23.69 ± 0.87^c^	24.13 ± 0.50^c^	23.65 ± 0.57^c^	2.79 ± 0.19^c^

Each value represents mean ± SEM for 9–10 mice. Different letters represent the statistical differences at *P* < 0.05 among the groups measured by Duncan’s test.

#### SDF improved oral glucose tolerance

**Fig. 1:**
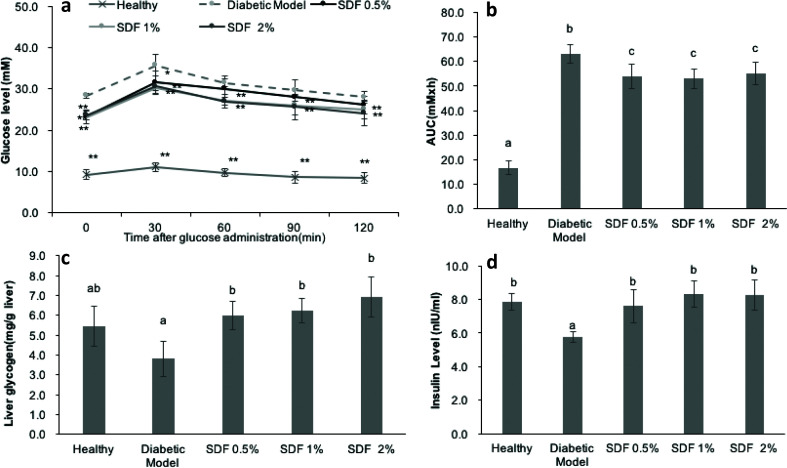
Effects of soluble dietary fiber (SDF) on glucose metabolism in mice: oral glucose tolerance test (OGTT) (a); calculated areas under the curve (AUC) from OGTT (b); liver glycogen content (c); and serum insulin levels (d). Each value represents mean ± SEM for 9–10 mice. In (a), statistical differences were evaluated using Student’s *t*-test. *P < 0.05 versus control group; **P < 0.01 versus control group. In (b–d), different letters represent the statistical differences at *P* < 0.05 among the groups measured by Duncan’s test.

As shown in the oral glucose tolerance test (OGTT) (Fig. 1a), glucose tolerance was impaired in the diabetic mice group. In contrast, SDF treatments at doses of 1 and 2% significantly suppressed the increase in blood glucose levels at all five time-points (*P* < 0.05). The mice treated with a low dose (0.5% of SDF) only showed significant suppression of the blood glucose level at 30 min (*P* < 0.05). The higher dose-treated mice had more potent glucose tolerance than the lowest dose-treated mice. As shown in Fig. 1b, the AUC value of the OGTT in the diabetic mice model was significantly higher than that of the healthy group. In comparison with the diabetic mice group, the AUC values of the SDF-treated groups were significantly decreased. The SDF-induced reductions in AUC values of the OGTT were comparable among groups with different doses.

#### SDF increased liver glycogen content

The liver is a crucial organ for the maintenance of glucose homeostasis, as it balances gluconeogenesis and glycogen synthesis. As shown in Fig. 1c, SDF treatment significantly enhanced liver glycogen storage in diabetic mice, which contributed to the suppression of hepatic glucose output and the improvement of glucose homeostasis.

#### SDF increased fasting insulin levels

The diabetic mice showed significantly lower fasting insulin levels than the healthy mice (Fig. 1d) as STZ injection destroys β-cells in the pancreas. The reduction in the insulin secretion led to a further increase in the FBG levels of the diabetic mice. The SDF-treated groups, at all doses, showed significantly increased levels of insulin compared with the diabetic mice group (*P* < 0.05), which may mean that SDF could help lower FBG.

### SDF improved the serum lipid profiles of diabetic mice

**Fig. 2:**
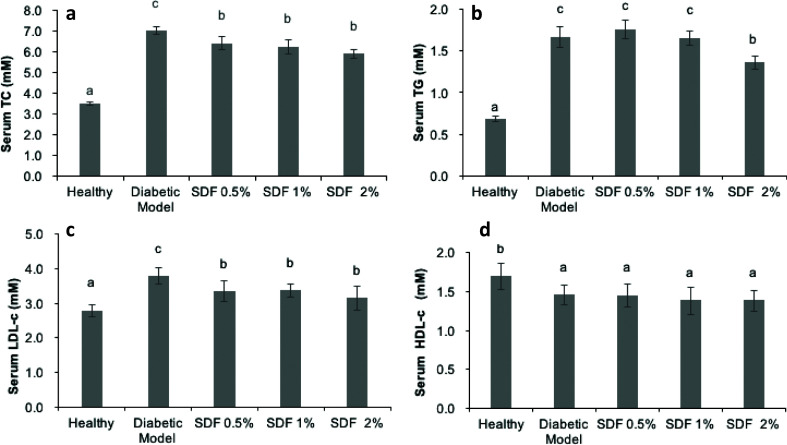
Effects of soluble dietary fiber (SDF) on the serum lipid profile in mice: total cholesterol, TC (a); total triglycerides, TGs (b); low-density lipoprotein cholesterol, LDL-c (c); and high-density lipoprotein cholesterol, HDL-c (d). Each value represents mean ± SEM for 9–10 mice. Different letters represent the statistical differences at *P* < 0.05 among the groups measured by Duncan’s test.

Diabetes is usually accompanied by hyperlipidemia. As shown in Fig. 2a–d, the diabetic mice showed higher serum levels of TC, TG, and LDL-c and lower levels of HDL-c in response to the high-fat diet. In comparison with diabetic mice, SDF interventions at doses of 0.5, 1, and 2% significantly reduced TC by 8.6, 11.2, and 15.8%, and LDL-c by 11.6, 10.9, and 16.7%, respectively (*P* < 0.05). SDF supplementation at a dose of 2% significantly reduced the TG level by 18.4% compared with the diabetic mice group (*P* < 0.05), while SDF supplementation at lower doses of 0.5 and 1% had no effects on TG.

### SDF attenuated hepatic lipid accumulation and high-fat diet/STZ-induced liver injury in diabetic mice

**Fig. 3:**
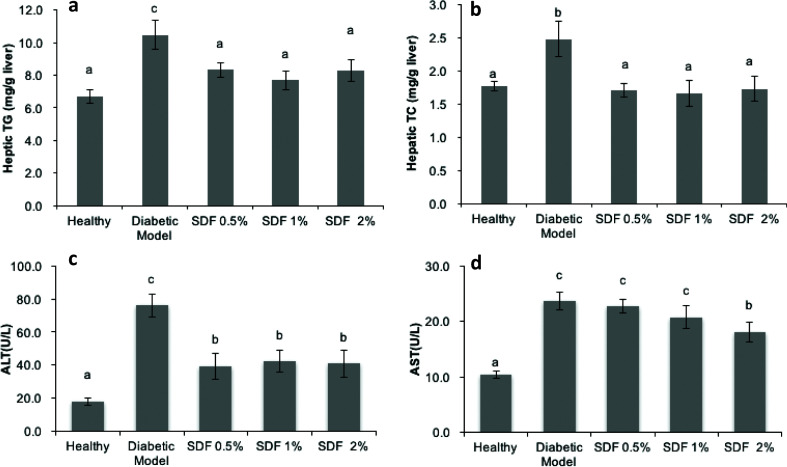
Effects of soluble dietary fiber (SDF) on lipid profiles and function parameters of liver in mice: hepatic TG (a); hepatic TC (b); serum alanine aminotransferase, ALT (c); and serum aspartate aminotransferase, AST (d). Each value represents mean ± SEM for 9–10 mice. Different letters represent the statistical differences at *P* < 0.05 among the groups measured by Duncan’s test.

As shown in Fig. 3a and b, the diabetic model had higher accumulated levels of hepatic TG and TC compared with the healthy group. A significant reduction in the hepatic TC and TG was observed in the SDF groups at all doses. Furthermore, glucose and lipid disorders may impair liver function in diabetic mice. ALT and AST levels are two useful biomarkers of hepatocellular injury. As shown in Fig. 3c and d, the diabetic mice groups had significantly higher ALT and AST levels than the healthy group (*P* < 0.05). Treatment with SDF at all doses resulted in significantly reduced ALT levels. Treatment with 2% SDF caused a significant decrease in AST by 21.7% (*P* < 0.05), while the treatments at doses of 0.5 and 1% caused no significant changes.

### SDF promoted the phosphorylation of AMPK in the liver of diabetic mice

**Fig. 4:**
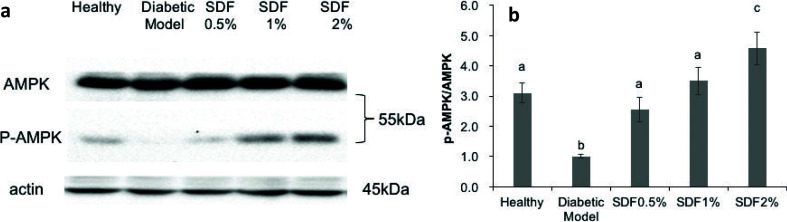
Protein expression of AMPK in the liver of mice by Western blot analysis: representative blots (a); the relative protein expression for signaling protein is shown as mean ± SEM (*n* = 4/group) and expressed as the fold change over the diabetic control (b). The results were all normalized to β-actin levels. Different letters represent the statistical differences at *P* < 0.05 among groups measured by Duncan’s test.

As shown in Fig. 4, the high-fat diet downregulated the phosphorylation of AMPK in the livers of diabetic mice by 67.8% (*P* < 0.05), which was consistent with previous reports (12) The administration of SDF at doses of 0.5, 1, and 2% substantially increased the phosphorylation of AMPK in the liver by 155.4, 249.8, and 357.7%, respectively. In addition, the level of hepatic AMPK phosphorylation in the 2% SDF group was higher than that in the healthy group (*P* < 0.05).

### SDF increased the SCFA contents in the cecum of diabetic mice

**Fig. 5:**
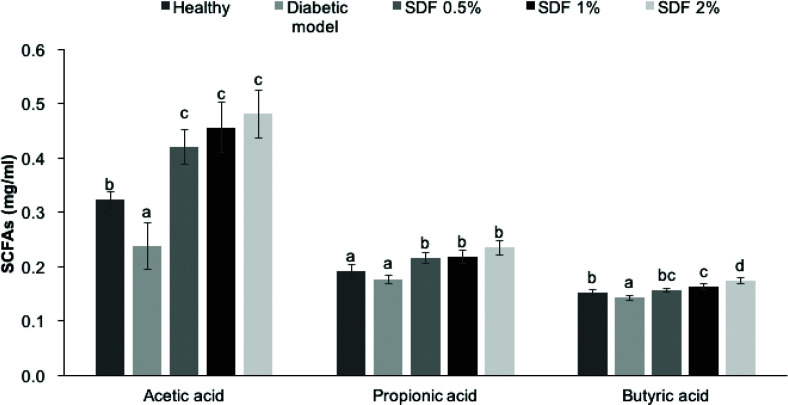
Effects of soluble dietary fiber (SDF) on cecal short-chain fatty acids in mice. Each value represents mean ± SEM for 9–10 mice. Different letters represent the statistical differences at *P* < 0.05 among the groups measured by Duncan’s test.

As shown in Fig. 5, compared with the healthy group, the high-fat diet significantly decreased the cecal SCFA content, especially acetic acid and butyric acid, by 25.2 and 5.7%, respectively (*P* < 0.05). The intake of SDF at doses of 0.5, 1, and 2% increased acetic acid levels by 76.3, 91.7, and 102.2%, respectively. Treatment with SDF at doses of 0.5, 1, and 2% resulted in higher production levels of propionic acid by 21.3, 23.1, and 32.64%, respectively. The concentrations of cecal butyric acid in the 0.5, 1, and 2% SDF groups increased by 9.8, 13.7, and 21.7%, respectively. Moreover, SDF interventions at all doses resulted in higher levels of acetic acid, propionic acid, and butyric acid than those of the healthy group (*P* < 0.05).

### Purification and structural analysis of polysaccharide from SDF

**Fig. 6:**
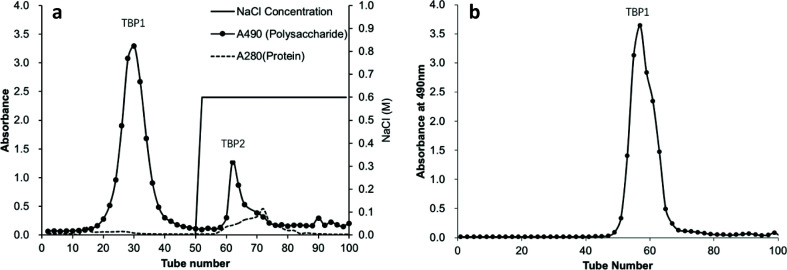
Chromatogram of eluted polysaccharide from soluble dietary fiber (SDF) on DEAE CL-6B column (a) and on Sepharose CL-6B column (b).

The SDF content in tartary buckwheat bran is 4.02 ± 0.19%. The SDF was purified by DEAE-Sepharose CL-6B column. As shown in Fig. 6a, two tartary buckwheat polysaccharide (TBP) fractions were detected, TBP1 and TBP2. The first peak TPB1 was the neutral polysaccharide and was the major polysaccharide fraction with higher polysaccharide content. Further purification of TBP1 using the Sepharose CL-6B column obtained a single polysaccharide peak (Fig. 6b). The protein and nucleic acids were not detected in TBP1. The purified TBP1 from SDF was used for further structural analysis.

The TBP1 was mainly composed of glucose (93.98%). Besides, it was composed of mannose (1.69%), galactose (1.48%), arabinose (1.37%), rhamnose (1.30%), and xylose (0.18%). The previous study showed that the polysaccharide from tartary buckwheat consisted of galactose, arabinose, xylose, and glucose with a molar ratio of 0.7:1:6.3:74.2(8). The TBP1 has the average molecular weight of 19.6 kDa, with polydispersity (Mw/Mn) of 1.129. It was found in the previous report that the molecular weight of polysaccharide from tartary buckwheat by Sephadex G-25 and G75 was 26 kDa (8).

**Fig. 7:**
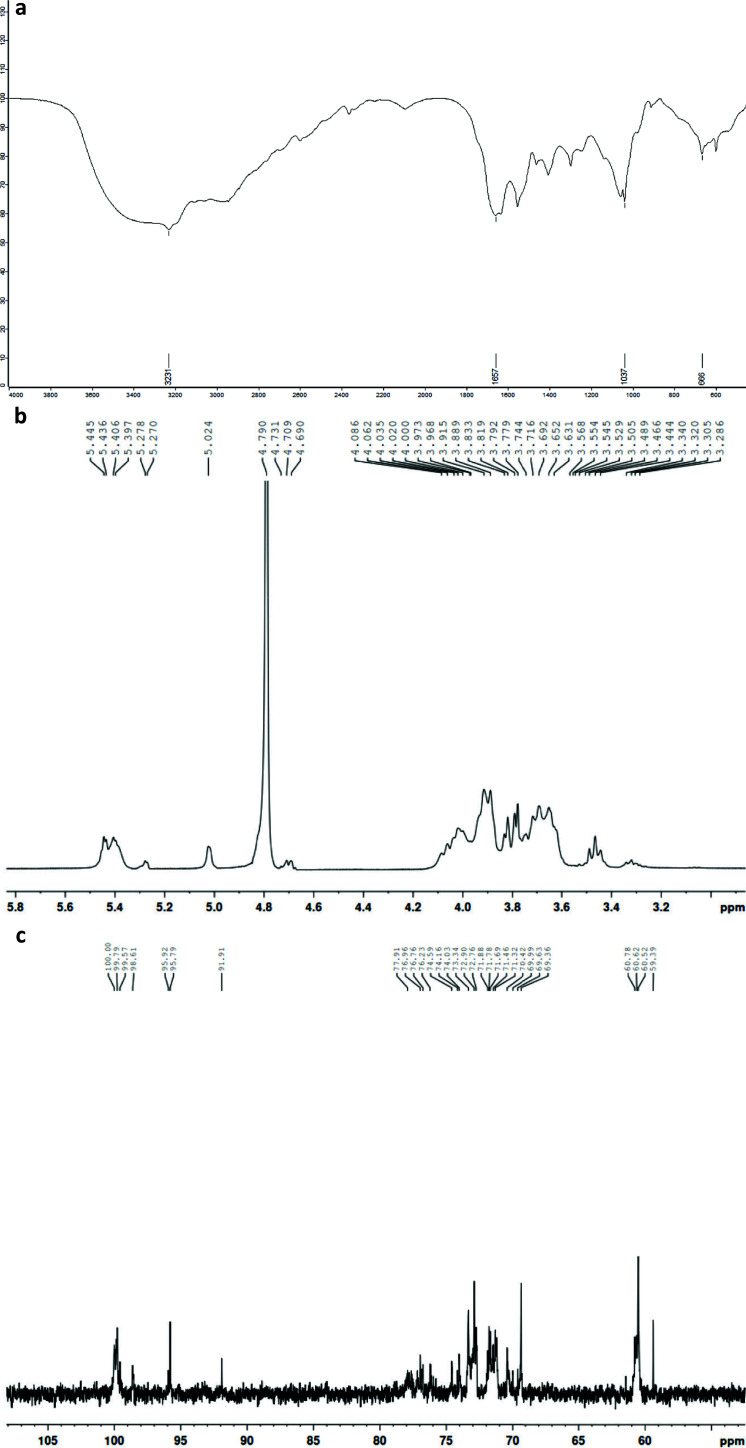

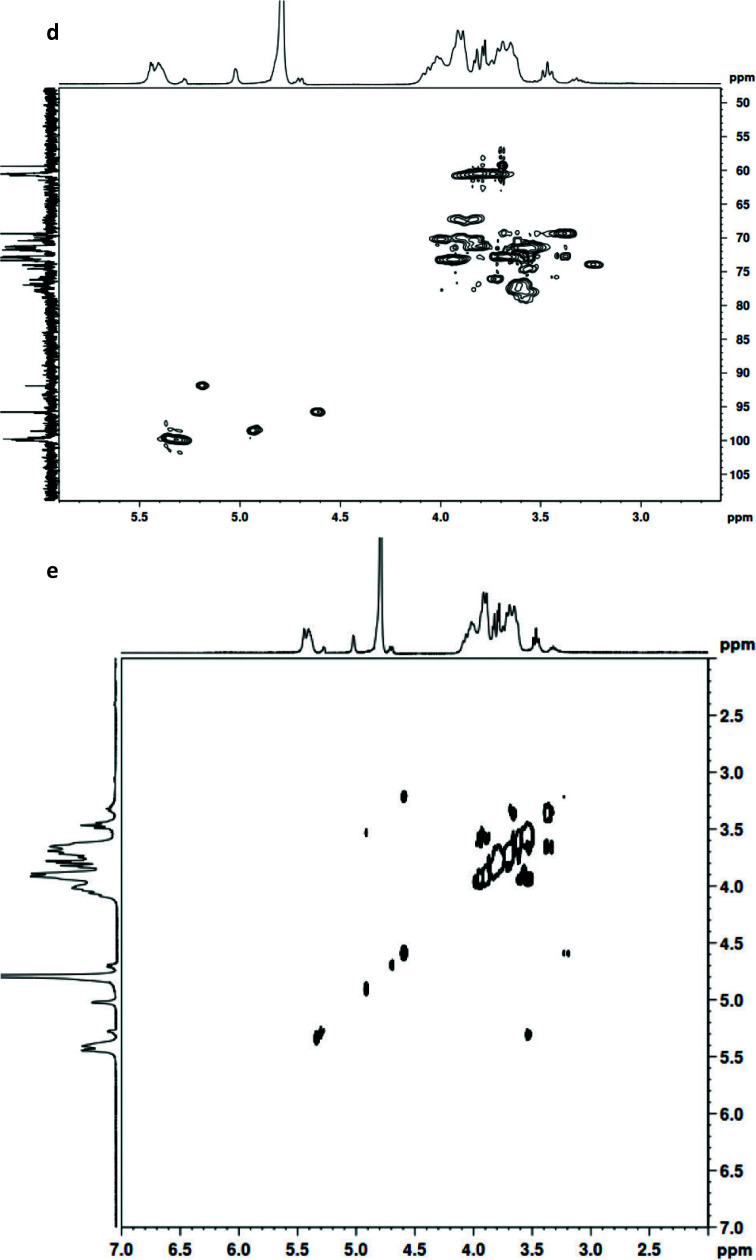
Structural characteristics of tartary buckwheat polysaccharide 1(TBP1) derived from FT-IR spectra (a) and NMR based on ^1^H NMR (b), ^13^C NMR (c), ^1^H-^13^C HSQC spectra (d), and ^1^H-^1^H COSY spectra (e).

As shown in Fig. 7a, the TBP1 displayed a typical FT-IR spectrum of polysaccharide (13). The broad intense characteristic peak at 3,387 cm^−1^ was attributed to the stretching vibration of O-H. The peaks of 2,929 cm^−1^ were ascribed to the C-H stretching (14). The peaks from 1,641 cm^−1^ and 1,415 cm^−1^ belong to C=O bending and C-H bending, respectively, which were typical infrared absorption peaks of polysaccharides (15). In addition, three stretching peaks between 1,154 cm^−1^ and 1,024 cm^−1^ indicated the presence of pyranoside. The weak band at 848 cm^−1^ and 928 cm^−1^ was ascribed to α-glycosidic bond and β-glycosidic bond in the polysaccharides, respectively (8, 15, 16).

The structural characteristics of TBP1 derived from NMR analysis are shown in Fig. 7b–e, namely, ^1^H, ^13^C NMR, ^1^H-^13^C HSQC, and ^1^H-^1^H COSY spectra. In general, the anomeric signals in ^1^H NMR were concentrated in the range of 4.69–5.45 ppm. The 5.02–5.45 ppm region represented the type of α-configuration, and the 4.69–4.73 ppm region represented the β-configuration. The anomeric signals in ^13^C NMR were in the low field ranging from 91.9 to 100.0 ppm, of which 91.9–98.6 ppm represented the type of α-configuration, and 99.6–100.0 ppm represented the β-configuration of glucose (17, 18). The maximum heap of anomeric signals among the regions δ 5.45–5.40 ppm in ^1^H NMR and δ 91.9, 99.6, 99.8, and 100.0 ppm in ^13^C NMR indicated that D-glucopyranose residues in TBP1 were mainly of the type α-configuration. The primary shift signal of C-1 at δ 99.8 and 99.6 ppm combined with C-2 shift at δ 70.4 ppm proved the dominant existence of α-D-Glcp-(1→linkage, which were probably at terminal position (19). The shift signal of C-1 at δ 100.0 ppm and C-6 at δ 69.4 ppm could prove the existence of →6)-β-D-Glcp-(1→linkage, respectively (20). The weak C-1 signal at δ 91.9 ppm indicated -α-D-Glcp-(4→linkage (21). The C-1 signal at δ 95.8 and 95.9 ppm with the C-2 signal at δ 74.0 ppm could be assigned to linkage →1)-β-D-Glcp-(6→. Both the C-1 signal at δ 98.6 ppm and the C-6 shift at δ 60.8 ppm demonstrated that →2)-β-D-Glcp-(1→ linkage existed, although H-2, H-3, H-4, and H-5 were hard to be distinguished due to signal overlapping (20). According to the literature above, major signals among the region δ 69.3-77.0 ppm could be attributed from C-2 to C-5, respectively. The chemical shifts of the characteristic signals in the ^1^H and ^13^C NMR spectra of TBP1 are summarized in Table 3. The structure of TBP1 mainly consisted of α-D-Glcp linkage.

**Table 3:** Chemical shifts of the characteristic signals in the ^1^H and ^13^C nuclear magnetic resonance spectra of TBP1

Glycosyl residues	Chemical shifts (ppm)					
	H_1_/C_1_	H_2_/C_2_	H_3_/C_3_	H_4_/C_4_	H_5_/C_5_	H_6_/C_6_
α-D-Glcp-(1→	5.40–5.45	3.60–3.65	nd	nd	nd	3.7, 3.8
	99.8–99.6	70.4	74.2	70.4	72.9	60.5
→6)-α-D-Glcp-(1→	5.40–5.45	3.60–3.65	nd	nd	nd	3.7, 3.8
	100.0	70.4	nd	nd	72.8	69.4
α-D-Glcp-(4→	5.27	nd	nd	nd	nd	3.6
	91.9	nd	74.0	77.9	71.3	60.8
→1)-β-D-Glcp-(6→	4.71	3.95	nd	nd	nd	nd
	95.8, 95.9	74.0	76.2	70.4	nd	nd
→2)-β-D-Glcp-(1→	5.40–5.45	nd	nd	nd	nd	3.7, 3.8
	98.6	72.7	74.0	70.4	72.8	60.8

## Discussion

In this study, the hypoglycemic and hypolipidemic activities of SDF from tartary buckwheat bran in high-fat diet and STZ-induced diabetic mice were investigated. In addition, it was found that the amelioration of glucose and lipid metabolism by SDF was accompanied by an increase in SCFAs in the cecum and phosphorylation of AMPK in the liver.

The glucose metabolism in diabetic mice was improved by SDF (Table 2 and Fig. 1), which was consistent with data on the hypoglycemic effects of SDF from other sources, such as oat β-glucan (22, 23), deoiled cumin (24), barley (25), and bamboo shoot shell (26). Similar to the results of this study, the lowering of FBG levels by oat β-glucan was accompanied by significantly increased levels of liver glycogen (23) and serum fasting insulin (22, 23). The *in vitro* study showed that the hypoglycemic effects of dietary fiber may be attributed to its ability to lower the postprandial glucose level by binding to glucose and preventing its diffusion (26, 27). An *in vitro* glucose absorption capacity test was performed by the research team of this study, which revealed that SDF has a glucose absorption capacity of 11.39–2070.64 μmol(glucose)/g(SDF) in different concentrations of glucose solution (10-200 mM) (detailed data are not shown in this study). In addition, the non-starch soluble polysaccharide from tartary buckwheat has been reported to inhibit α-glucosidase activity, which indicates potential hypoglycemic effects (8).

Furthermore, SCFAs, such as acetic, propionic, and butyric acids, are major metabolites produced by the intestinal fermentation of complex carbohydrate fibers by the microbiota. In this study, SDF enhanced SCFA production in the cecum (Fig. 5), which resembled the effects of SDF from cumin (24). The levels of SCFAs in the SDF groups exceeded those in the healthy group. SCFAs can enter the systemic circulation and activate AMPK in intestinal epithelial (28), skeletal muscle (29), and liver cells (30) either directly or indirectly. It is suggested that activation of AMPK through increased SCFA production is the main mechanism underlying the beneficial effects of a high-fiber diet on metabolic syndrome (31–34). In agreement with previous studies (32–34), SDF treatment not only resulted in SCFA production but was also accompanied by the activation of AMPK in the liver (Figs. 4 and 5). The phosphorylation of AMPK functions as a major cellular fuel gauge and a master regulator of glucose and lipid metabolism through its effects on energy metabolism (28, 35–38, 39).

AMPK activation can lead to hyperglycemia in diabetic mice (Figs. 1 and 5). Hepatic gluconeogenesis appears to be suppressed by SCFAs, such as acetic acid (36, 40) and propionic acid (41), through upregulation of AMPK. This aligns with the results of this study, in which increased glycogen storage by SDF supplementation was observed, and this may indicate that the effects of SDF are mediated by hepatic AMPK activation (38, 42).

Furthermore, SDF treatment ameliorated the lipid profiles in both serum and liver (Figs. 2 and 3), which aligns with data on the hypolipidemic effects of SDF from cumin (24), soybean residue (43), and guar gum (44). The SCFAs that were absorbed and metabolized in the liver may be involved in lowering plasma cholesterol levels (45). The results of this study revealed that the highest dose (2%) of SDF was more effective at improving lipid metabolism, which may be related to the dose-dependent effects of SDF on butyric acid (Figs. 2 and 5). In addition, the reduction in hepatic lipid accumulation may be related to SCFA-induced AMPK activation, as reported previously (30, 37, 46, 47). Furthermore, the result of this study revealed that SDF effectively protected against high-fat diet-induced liver injury (Fig. 3), similar to pectin (48) and SDF from barley (25). As the accumulation of hepatic lipid is less modifiable than that of serum lipid, SDF may not have any significant effects on hepatic lipid accumulation.

Interestingly, this study showed that the dosage effects of SDF were not linearly linked with the hypoglycemic effects, which resembled the effects of SDF from other sources (22, 24, 49). As reported previously, the SDF doses from 0.5 to 2% might reach the activity plateau. The hypoglycemic effects of dietary fiber were mainly through binding glucose and/or digestive enzyme so as to inhibit glucose release and absorption. SDF from tartary buckwheat reached its highest inhibition activity plateau against α-glucosidase with a relatively low concentration of 0.8 mg/mL (8). In addition, the hypoglycemic and hypolipidemic activities of SDF are indirectly affected by SCFAs.

Tartary buckwheat contains several hypoglycemic and/or hypolipidemic compounds, such as rutin, fagopyritols, D-chiro-inositol (DCI), and protein (50). Rutin has been shown not to affect serum TC (51), ALT, and AST (52) in high-fat diet-fed rats. In addition, rutin has little effect on SCFA production (51). However, significant reductions in the serum TC and LDL-c levels of patients with type 2 diabetes were observed in the tartary buckwheat intervention group (6). This study revealed that SDF might contribute to the SCFA production and reduction of TC, similar to tartary buckwheat protein (53). In addition, as tartary buckwheat contains several bioactive compounds, the synergistic effects of these compounds need to be analyzed to reveal the mechanisms of action of tartary buckwheat in the process of glucose and lipid metabolism. Furthermore, the SDF from tartary buckwheat could be used as a novel source of dietary fiber to assist in the amelioration of glucose and lipid metabolism. As pseudo-cereals are a good source of dietary fiber, further studies that compare the anti-diabetic properties of SDFs with different pseudo-cereals will benefit the exploration of functional dietary fibers.

The mainly effective component of SDF is a neutral polysaccharide, TBP1, which mainly consisted of α-D-Glcp linkage (Table 3 and Fig. 7). The previous study found that the backbone of TBP was composed of (1→4)-linked -D-Glcp (8). It suggests that the structure of SDF from tartary buckwheat is different from that of other bioactive soluble polysaccharides obtained from some cereals and pseudo-cereals in common, such as arabinoxylans and β-glucans, which are connected by β-configuration (54). The difference in polysaccharide structure among these cereals and pseudo-cereals probably results in the differences in their intestinal function and the indirect interference of glucose and lipids metabolism, which still need to be further illustrated.

## Conclusion

This study confirms the *in vivo* hypoglycemic and hypolipidemic effects of SDF from tartary buckwheat and demonstrates the correlation between an increase in SCFA excretion and the promotion of hepatic AMPK phosphorylation. The collective findings reveal the physiological activities of SDF from tartary buckwheat, which contribute to the mechanisms by which tartary buckwheat improves glucose and lipid metabolism. In addition, the SDF of tartary buckwheat could be used as a new source of dietary fiber for the treatment of diabetes.
